# PGLYRP-1 mediated intracellular peptidoglycan detection promotes mucosal protection

**DOI:** 10.21203/rs.3.rs-5118704/v1

**Published:** 2024-10-14

**Authors:** Shuyuan Chen, Rachel Putnik, Xi Li, Alka Diwaker, Marina Vasconcelos, Shuzhen Liu, Junhui Zhou, Lei Guo, Lin Xu, Sebastian Temme, Klare Bersch, Stephen Hyland, Jianyi Yin, Ezra Burstein, Jeffrey C. Gildersleeve, Catherine Leimkuhler Grimes, Hans-Christian Reinecker

**Affiliations:** 1 Department of Medicine, Division of Digestive and Liver Diseases, University of Texas Southwestern Medical Center, 5959 Harry Hines Boulevard, Dallas, Texas 75390, United States; 2 Department of Chemistry and Biochemistry, University of Delaware, Newark, Delaware 19716, United States National Cancer Institute.; 3 Quantitative Biomedical Research Center, Peter O’Donnell Jr. School of Public Health, University of Texas Southwestern Medical Center, United States; 4 Chemical Biology Laboratory, Center for Cancer Research, National Cancer Institute, Frederick, MD, 21702, United States; 5 Department of Immunology, Center for the Genetics of Host Defense, and Simmons Cancer Center University of Texas Southwestern Medical Center, 5959 Harry Hines Boulevard, Dallas, Texas 75390, United States

## Abstract

Peptidoglycan recognition proteins (PGRPs or PGLYRPs) are implicated in the control of the intestinal microbiota; however, molecular requirements for peptidoglycan (PGN) binding and receptor signaling mechanisms remain poorly understood. We identified PGLYRP-1 as a receptor for the disaccharide motif of lysine N-acetylglucosamine N-acetylmuramic tripeptide (GMTriP-K) with a newly constructed PGN microarray. Surprisingly, PGLYRP-1 was required for innate immune activation of macrophages by GMTriP-K but not N-acetylglucosamine N-acetylmuramic dipeptide (GMDiP) or muramyl dipeptide (MDP). In macrophages, intracellular PGLYRP-1 complexed with NOD2 and GEF-H1, both of which were required for GMTriP-K-regulated gene expression. PGLYRP-1 localized to the endoplasmic reticulum and interacted at the Golgi with NOD2 upon GMTriP-K stimulation. PGLYRP-1 upregulation and its dependent gene expression signatures were induced in both mouse intestinal inflammation and human ulcerative colitis. Importantly, PGLYRP-1 activation by GMTriP-K resulted in innate immune activation and protection of mice from colitis. Our results show that PGLYRPs can function as intracellular PGN pattern recognition receptors for the control of host defense responses in the intestine.

## Graphical abstract |

**PGLYRP-1 mediated recognition of peptidoglycans in macrophages controls mucosal inflammation** PGLYRP-1 is required for detection of intracellular PGN containing disaccharide tripeptides PGLYRP-1 localizes to the endoplasmic reticulum and interacts with NOD2 and GEF-H1 in the Golgi apparatus to induce the expression of immune regulatory cytokines, chemokines, and metabolic regulators that protect from intestinal inflammation.

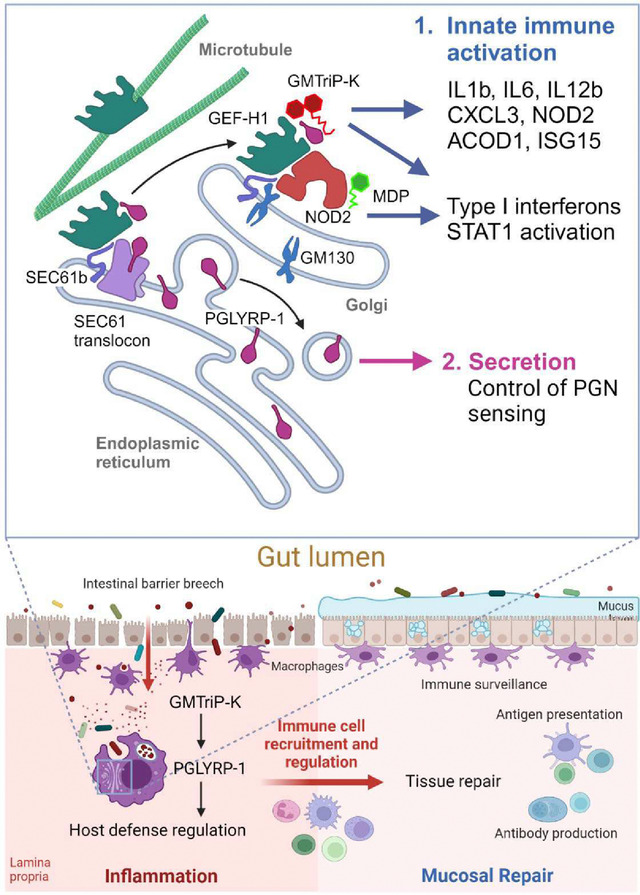

## Introduction

The innate immune system recognizes molecular patterns of microorganisms to trigger protective immune responses and genetic variants that impair immune recognition of peptidoglycans (PGN) have been linked to inflammatory bowel disease ^1^. Peptidoglycan recognition proteins (PGRPs) play an important role in innate immunity by recognizing PGN and they are evolutionarily conserved pattern recognition receptors in both invertebrates and vertebrates ^2,3^. Humans and mice have four PGRPs, namely PGLYRP-1, PGLYRP-2, PGLYRP-3, and PGLYRP-4 ^2,3^. PGLYRPs, 1, 3 and 4 are bactericidal and PGLYRP-2 has amidase activity when secreted ^4^. How PGRPs modulate immune function is incompletely understood ^5,6^ and no direct signaling mechanism have been established for vertebrate PGRPs compared to invertebrate PGRPs ^7,8^. Instead, NOD-like receptors (NLRs) NOD1^9^ and NOD2 ^10,11^ have been linked the recognition of PGN fragments and were found to activate innate immune responses through mechanisms that require guanine nucleotide exchange factor (GEF)-H1 for signaling ^12–14^.

**Fig. 1 |:**
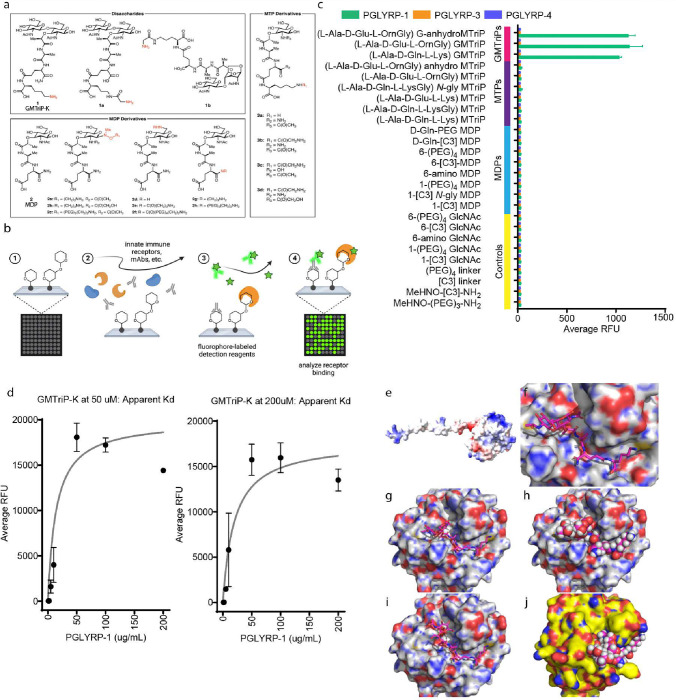
PGLYRP-1 specifically binds GMTriP-K **a,** Peptidoglycan fragment library: GMTriP-K (1) and MDP (2); synthetic peptidoglycan fragments (1a-b, 2a-h and 3a-d) were all prepared with amine linkage points for attachment to the array surface via NHS-chemistry. **b,** General workflow for the printing, incubation, and analysis of the PGN small fragment microarray. **c,** Microarray binding of PGLYRPs. PGLYRP-1 binds to the disaccharide components of the array, whereas PGLYRP-3 and PGLYRP-4 show no association with the compounds on the array. **d,** Determination of Apparent Dissociation Constant for PGLYRP-1 to GMTriP-K. **e,** Alphafold protein structure prediction of human PGLYRP-1. **f-I,** Docking prediction of GMTriP-K to human PGLYRP-1. **j,** Docking prediction of PGLYRP-3 and GMTriP-K demonstrating shallow GlcNAc preventing interaction. For synthetic procedures and compound characterization (NMRs, HRMS), please see the SI. For **(c)**, each condition was screened in at least biological triplicate and technical replicated (see SI for raw image files and other biological replicate binding data).

The PGN core repeating unit includes a disaccharide unit (GlcNAc-MurNAc) with short peptides linked to the lactyl group of the muramic acid (Fig. 1a). PGN incorporates diversified elements across species, including sugar modification, use of rare amino acids and variable cross-linking chemistries connecting adjacent peptide chains ^15^. Furthermore, PGN can be broken down by host/bacteria lysozymes and transglycosylases and the resulting diverse PGN repertoire poses challenges for immune recognition requiring specific receptors to initiate the host defenses ^16^. To examine the role of PGN chemical structure on immune activation, we established synthetic strategies for PGN fragments with a variety of pathogenic relevant features ^17^. These studies identified N-acetylglucosamine N-acetylmuramic tripeptide (GMTriP-K) as a unique immune effector inducing host defense responses distinct from the model compound muramyl dipeptide (MDP) ^17^. However, it remained unclear how GMTriP-K and MDP, which differ from each other in one sugar unit (GlcNAc) and one peptide (lysine), could induce such distinct immune responses. We hypothesized that additional PGN receptors may be involved in specifying immune responses to these diverse PGN derivatives. To identify such PGN host receptor(s) and determine their binding specificities, we developed a small-fragment microarray incorporating a wide variety of existing and novel glycan and peptide chain modifications into the PGN backbone. This approach allowed the identification of a previously unknown intracellular receptor system for GMTriP-K responsible for regulating specific transcriptional responses different from other PGN for the control of intestinal inflammation.

### PGLYRP-1 binds the disaccharide motif of GMTriP-K

GMTriP-K represents the main disaccharide fragment generated via lysozyme digestion of the cell wall ^17^ and 1,6-anhydro disaccharide is the major lytic product produced by bacteria during bacterial growth and division ^18^. To identify the PGN-immune receptors mediating the recognition of GMTriP-K, we synthesized an expanded library of PGN derivatives and printed them on a microarray surface using NHS-activation chemistry (Fig. 1b). The dynamic assay surface was optimized for downstream screening applications and contained biologically relevant modifications of mono- and disaccharides in different orientations, positioning around the carbohydrate core and extending peptides (Fig. 1c). As the focus of this study was disaccharide fragments, GMTriP-K was immobilized to the array surface through the free amine on the extending lysine sidechain, exposing the GlcNAc-MurNAc disaccharides. In addition, the canonical 1,6-anhydro disaccharide motif found in the pertussis-derived tracheal cytotoxin was included with an ornithine modification on the third peptide ^19^. The arrays were designed and included controls, such as GlcNAc, linkers, and modifications found within the peptidoglycan (see Tab. S1 for a full list of array components and the supporting information for molecular details) to ensure comprehensive binding of potential immunoglobulins or innate immune proteins. Prior to use, the array was fully validated using a variety of antibodies and lectins (see Fig S8–10 and supporting information for a complete description of the validation methods).

The validated PGN array was then used to evaluate the binding preferences of three bactericidal PGLYRPs: PGLYRP-1, PGLYRP-3, and PGLYRP-4 (Fig. 1c). When PGLYRP-1 (10 μg /mL) was applied to the microarray, the data showed that the innate immune receptor bound to GMTriP-K and anhydro-containing disaccharide tripeptides (Fig. 1c, Figs. S9–11). However, no binding was observed for monosaccharide-containing units (Fig. 1c). When a concentration gradient of the receptor was applied to the array (1 μg /mL – 200 μg /mL), affinity to the disaccharide fragments was observed as low as 2 μg/mL, suggesting that the receptor is a μM binder for GMTriP-K (Fig. 1d). Fitting these data using a saturation binding experiment, the apparent Kd for GMTriP-K to bind PGLYRP-1 was estimated to be 5.7 – 42.2 μM (Show in Fig. 1d, supplementary text and Fig. S12). Furthermore, no binding was observed when PGLYRP-3, and PGLYRP-4, were applied to the array at these concentrations (1 μg/mL – 200 μg/mL) (Fig. 1c). It should be noted that the PGN array presented PGN fragments in multiple orientations, with some fragments anchored to the peptide (compounds 1a-c, 2g–h, and 3a-d), and others anchored to the carbohydrate (2a-f), Tab. S1). However, PGLYRP-1 was only bound to the components of the array when the disaccharide moiety was present and exposed, suggesting that PGLYRP-1 binds preferentially to disaccharide moieties over the monosaccharide-only residues.

We next modeled the binding of human PGLYRP-1 (Fig. 1e) with GMTriP-K. The minimized structure of GMTriP-K was modeled after the known crystal structure of PGLYRP-3 complexed with a MTP ligand (pdb code 1TWQ) as PGLYRP-3 is 44% identical and 68% similar to PGLYRP-1. Using these coordinates, the GMTriP-K was docked using AutoDock Vina and minimized into a model of PGYRP-1 (obtained from AlphaFold) using the Webina and YASARA servers ^20^. The full GMTriP-K ligand was constructed using the programs PyMOL and ChemDraw3D. The final modeled structure aligned well with our experimental data for PGLYRP-1 ligand interactions. We found the lysine solvent exposed multiple hydrogen bonds both in the peptide and the MurNAc portion of the fragment, and a perfectly sized binding pocket for the GlcNAc, permitting many van der Waals interactions. Particular attention was given to the stereochemistry of the reducing end of the disaccharide, assuring that the hydroxy group was in the minimal beta confirmation (Fig. 1f–h). PGLYRP-1 binding was prevented when the alternative chirality at the C1 position was used, removing a hydrogen bond and rotating the neighboring GlcNAc residue backwards into a sterically crowded region of the protein (Fig. 1i). Alternatively, in PGLYRP-3 models, the pocket for the GlcNAc was too shallow to dock GMTriP-K (Fig. 1j), agreeing with the array data, which does not yield a GMTriP-K-PGLYRP-3 interaction. Together, the PGN fragment array data and ligand receptor modeling analysis indicated that PGLYRP-1 can specifically bind to the disaccharide units of GMTriP-K.

### PGLYRP-1 is required for GMTriP-K regulated gene expression

**Fig. 2 |:**
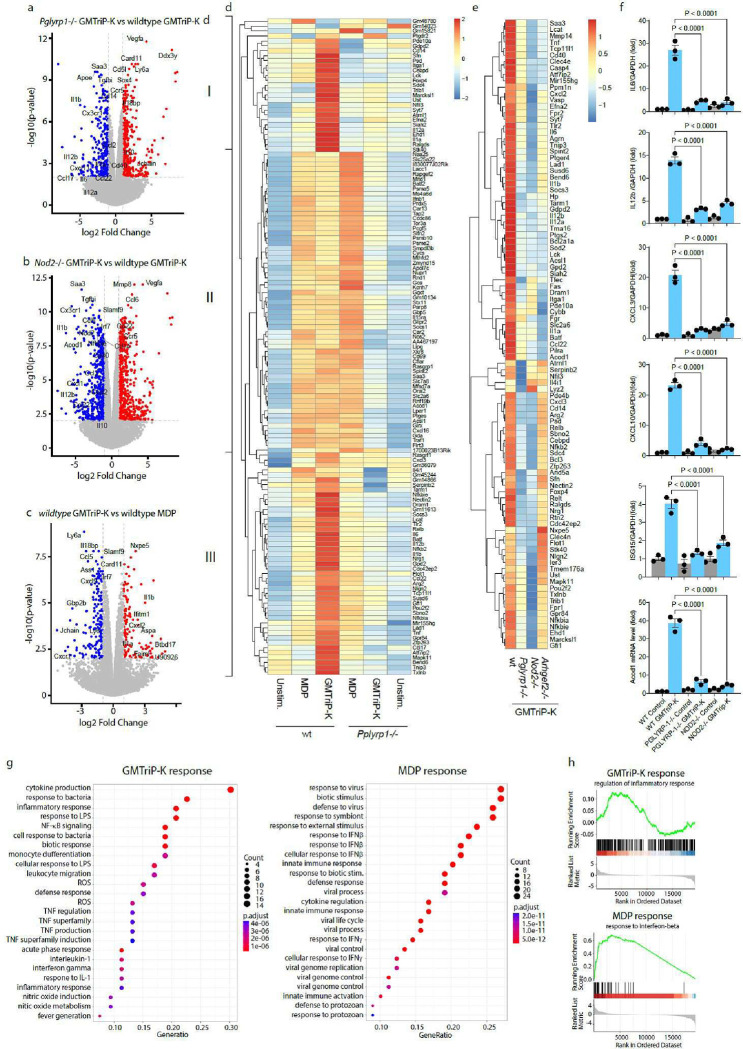
PGLYRP-1 is required for GMTriP-K-mediated transcriptional regulation in the NOD2/GEF-H1 pathway **a,** RNA-seq and volcano blot analysis of gene expression in BMDMs from wild type and *Pglyrp1*−/− mice after 18 hours of stimulation with 25 μM GMTriP-K. **b,** Volcano blot analysis of gene expression in BMDMs from wild type and *NOD2*−/− mice after 18 hours of stimulation with 25 μM GMTriP-K. **c,** Volcano blot analysis of gene expression in BMDMs from wild type mice after 18 hours of stimulation with 25 μM GMTriP-K or 25 μM MDP. **d,** Hierarchal cluster analysis of genes induced by either stimulus in wild type or *Pglyrp1*−/− BMDMs. **e,** Hierarchical cluster analysis of genes induced by GMTriP-K in BMDMs from wild type, *Pglyrp1*−/−, *Nod2−/−*, or *Arhgef2*^*−/−*^ mice. **f,** pPCR analysis of gene expression in BMDMs from indicated mouse strains stimulated with 25 μM GMTriP-K or 25 μM GMDiP stimulation on BMDMs from wild type and *Pylyrp-1−/−* mice for 18 hours. **g, h,** Gene set enrichment analysis of the differentially expressed genes induced by indicated stimulus in wild type BMDM. **g,** Top 25 enriched signaling pathways for the differentially expressed gene sets. **h,** Gene set enrichment analysis summarized in mountain plots representing significantly enriched (left) or depleted (right) of genes for the indicated gene sets and collections. Experiments were repeated at least twice.

To determine whether PGLYRP-1 is required for the induction of innate immune responses by GMTriP-K, we performed RNA-seq analysis of wild type and PGLYRP-1 deficient macrophages. GMTriP-K stimulation regulated significantly 3435 (p<0.05) (Tab. S2) genes in wildtype macrophages and 2500 (p<0.05) genes in *Pglyrp1*−/− macrophages after 18-hour stimulation (Tab. S3). Compared to wild type macrophages, *Pglyrp1*−/− macrophages expressed significantly less GMTriP-K induced immune regulatory genes such as Il1a, Il1b, Il12b, Saa3, Cx3cr1, Ccl2 (Fig. 2a, Tab. S4) indicating that PGLYRP-1 was required for the transcriptomic response to GMTriP-K. Remarkably, compared to wild type BMDMs, the expression of same gene signature including Il1a, Il1b, Saa3 and Il12b was also significantly reduced in *Nod2*−/− macrophages upon GMTriP-K stimulation, indicating that PGLYRP-1 functions as a GMTriP-K receptor in the NOD2 pathway ( Fig. 2, Tab S5) .

We next determined whether GMTriP-K induced immune responses differed from those induced by MDP in wild type, *Pglyrp1*−/− *Nod2*−/− and *Arhgef2*−/− macrophages. To better understand PGLYRP-1-dependent gene regulation, we performed hierarchical clustering of differentially regulated genes (4-fold difference) by GMTriP-K or MDP in wildtype and *Pglyrp1*−/− macrophages (Fig. 2d). We found that PGLYRP-1 was required for GMTriP-K but not MDP-induced gene expression (Fig. 2d, Tab. S6). Three main clusters emerged from this analysis in which PGLYRP-1 was required for gene induction by GMTriP-K. Cluster 1 represented genes such as *Il1a, Il12a, Cd14, Stk40, Itga1,* and *Foxp4*, which depended on PGLYRP-1 expression and were specifically induced by GMTriP-K but not by MDP (Fig. 2d). Cluster 2 contained genes, such as *Ifnb, Acod1, Cxcl16, Nos2*, and *Gbp5* whose expression was increased in PGLYRP-1-deficient compared to wild type macrophages in response to MDP. Cluster 3 contained genes associated with inflammation, such as *Il6, Il1b, Tnf, Relb, and Il12b,* which required PGLYRP-1 and these genes were induced at significantly higher levels with GMTriP-K stimulation compared to MDP (Fig. 2d).

We next determined whether PGLYRP-1 dependent proinflammatory gene expression required NOD2 and GEF-H1. Among genes uniquely induced by GMTriP-K (vs. MDP) in wild type BMDMs, we selected genes with a z-score >2 (Tab. S7). This gene list was used for hierarchical clustering in stimulated wild type and PGLYRP-1-, NOD2-, and GEF-H1-deficient BMDMs (Fig. 2e). This approach revealed that the GMTriP-K-induced gene expression required PGLYRP1, NOD2 and GEF-H1 for the induction of inflammatory regulators such as Tnf, Il6, Il1a, Il1b, Il12a, and Il12b (Fig. 2e). We also confirmed in independent experiments that the induction of key immune and metabolic regulators such as Il6, Il12b, Cxcl3, Cxcl10, Acod1 and Isg15 by GMTriP-K required PGLYRP-1 and NOD2 (Fig. 2f).

Functional enrichment analysis confirmed that GMTriP-K induced immune responses were significantly different from those induced by MDP. GMTriP-K-induced signatures were significantly enriched for “positive regulation of cytokine production” and “response to molecules of bacterial origin” (Fig. 2g). In contrast, MDP responses were associated with type I interferon responses, as indicated by the “response to interferon beta” and “response to virus” pathway association (Fig. 2g). Enrichment analysis showed a correlation between GMTriP-K responses and the regulation of an inflammatory response, while the MDP response was best characterized as a response to IFN-β (Fig. 2h).

To better understand MDP specific transcriptional response in PGLYRP-1 deficient macrophages, we selected genes with a z-score >2 preferentially induced by MDP (vs. GMTriP-K) in wild type BMDMs (Tab. S8). MDP responses were indeed characterized by a type I interferon signature that included members of the GBP and SLFN gene families, Oasl1/2, Rsad1, Cxcl10, Isg15, Irf7, Stat2, and Zbp1 (ED Fig. 1a). (ED Fig. 1a). Hierarchical clustering revealed that MDP-induced gene expression was independent of PGLYRP-1 but required NOD2 and GEF-H1(ED Fig. 1a). Surprisingly, MDP-induced gene expression increased significantly in the absence of PGLYRP-1 compared to wild type BMDMs (ED Fig. 1a).

We thus investigated type I interferon receptor activation after GMTriP-K and MDP stimulation. We found that GMTriP-K as well as MDP induced activation of STAT1 (ED Fig. 1b). However, PGLYRP-1 was specifically required for the induction of STAT1 phosphorylation by GMTriP-K, but not by MDP (ED Fig. 1b). In the absence of PGLYRP-1 we noted an increased phosphorylation of STAT1 by MDP (ED Fig. 1b). Both PGN required NOD2 and GEF-H1 for STAT1 activation (ED Fig. 1b, c). This was dependent on the induction of type I interferons because Ifnar1−/− mice did not respond with STAT1 activation when stimulation with either PGN (ED Fig. 1d). GEF-H1 was required for PGN-induced type I interferon induction but not for IFN receptor signaling, as IFNβ or IFNγ mediated STAT1 phosphorylation was absent in *Arhgef2*−/− deficient macrophages (ED Fig. 1e).

To confirm the specificity and requirement of PGLYRP-1 for specific GMTriP-K induced signaling, we also stimulated macrophages with GMDiP. Indeed, PGLYRP-1 was not required for the induction of Il1a, Il6, Il12b, and Cxcl10 by GMDiP, consistent with the inability of GMDiP to bind to PGLYRP-1 in our microarrays (ED Fig. 1f).

Finally, phosphorylation of muramyl peptides by *N*-acetylglucosamine kinase (NAGK) is required for NOD2 activation ^21^; therefore, we assessed if NAGK had a role in GMTriP-K activation. We found that NAGK did not phosphorylate disaccharides but was able to phosphorylate monosaccharide controls (Fig. S13).

In summary, the gene expression and signaling analyses identified PGLYRP-1 as a specific receptor for GMTriP-K. PGLYRP-1 signals through NOD2 and GEF-H1 both of which are required for the induction of GMTriP-K specific transcriptional responses.

### PGLYRP-1 is expressed in the ER and Golgi apparatus

**Fig. 3 |:**
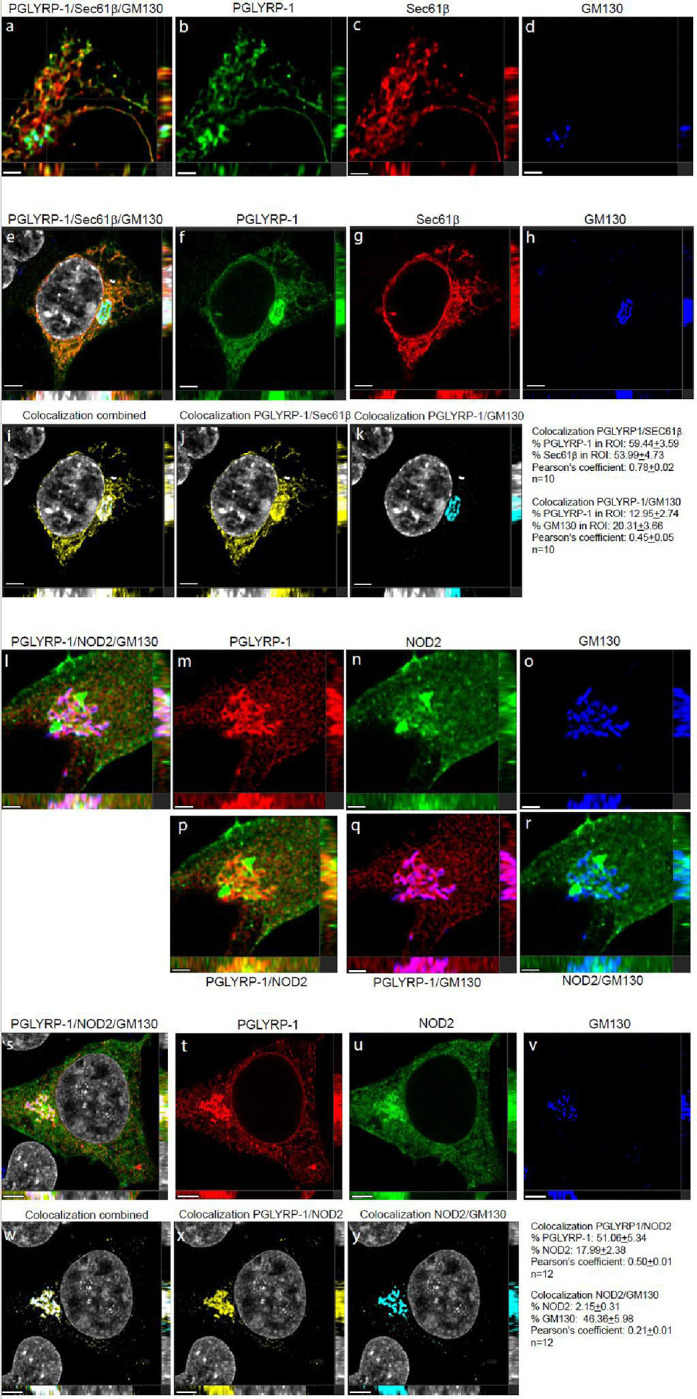
PGLYRP-1 localizes to the ER and Golgi **a-k**, Representative confocal microscopy images of HEK 293T cells expressing untagged (UT) or Flag-tagged PGLYRP-1, orange fluorescent protein (OFP)-tagged Sec61β and GFP-tagged NOD2. Cells were transfected with PGLYRP-1-UT **(a-d)** or PGLYRP-1-Flag **(e-k)** and Sec61β-OFP, and then probed with PGLYRP-1 and GM130 antibodies. PGLYRP-1, Sec61β-OFP and GM130 were detected in Alexa Fluor 488 (green pseudo-color), mOrange (red) and Alexa Fluor 647 (blue) channels, respectively. **l-y,** Cells were transfected with PGLYRP-1-UT and NOD2-GFP, and then stained for PGLYRP-1 and GM130. NOD2-GFP, PGLYRP-1 and GM130 were detected in EGFP (green pseudo-color), Rhodamine Red-X (red) and Alexa Fluor 647 (blue) channels, respectively. All channels were scanned sequentially, and images are pseudo-colored independent of channel wavelengths with nuclear counterstain in greyscale (bars indicate 2μm in **a-d, l-r** and 5μm in **e-k, s-y**). Each imaged cell typically represents 12 independent experiments with 10–12 cells analyzed for each condition.

We next aimed to identify the subcellular localization of PGLYRP-1 and associated signaling components. Surprisingly, PGLYRP-1 colocalized with Sec61β in the endoplasmic reticulum (ER) throughout the cell (Figs. 3a–c). Sec61β is a subunit of the Sec61 protein translocon complex expressed in the ER and Golgi. PGLYRP-1 was also identified in Golgi-associated structures near the cell nucleus positive for the cis-Golgi matrix protein 130 (GM130/GOLGA2) (Figs. 3 a,b,d). In addition, PGLYRP-1 localized to a vesicular compartment but not to cell surfaces (Figs. 3a–c). We analyzed the colocalization of PGLYRP-1 with ER and Golgi markers by confocal microscopy in 10 cells with one representative analysis shown (Fig. 3e–k). Analysis of the co-localization found significant overlap between PGLYRP-1 and the ER/Golgi markers in both the microscopy images and in volume analysis (Figs. 3i–k). 59.44±3.59% (Mean±SEM%) of the detected PGLYRP-1 overlapped with 53.99±4.73% of detected Sec61β in the cells with a mean Pearson’s coefficient of 0.779. 12.95±2.74% of the PGLYRP-1-positive compartment colocalized with 20.31±3.66% of the GM130-positive compartment with a mean Pearson’s coefficient of 0.45 (Figs. 3e–k, Tab. S9). We also generated colocalization channels based on the volume analysis to better visualize the ER- and Golgi-associated subcellular compartments that contain PGLYRP-1 (Fig. 3i–k).

We next determined the colocalization of NOD2 with cellular compartments that contained PGLYRP-1 or GM130 (Fig. 3l–r). In these experiments, we expressed untagged PGLYRP-1 and GFP-tagged NOD2 in HEK 293T cells and stained for PGLYRP-1 and GM130 (Figs. 3l–r). NOD2 was found to be expressed throughout the cytoplasm but enriched in the area of the Golgi. (Figs. 3l–o). Confocal image and volume analysis showed significant overlap of the aggregation of NOD2 with PGLYRP-1 in a GM130-positive compartment close to the nucleus (Figs. 3l–o). We found that 17.99±2.38% of NOD2 overlapped with 51.06±5.34% of the PGLYRP-1-positive compartment with a mean Pearson’s coefficient of 0.5 (n=12) (Fig. 3m, Tab. S10). In contrast, 2.15±0.31% of NOD2 colocalized with 46.36±5.98% of the GM130-positive compartment with a mean Pearson’s coefficient of 0.21 (Fig. 3s–y). The colocalization channels based on the confocal volume analysis for PGLYRP-1, NOD2 and GM130 showed that PRLYRP-1 and NOD2 enriched at the GM130-positive Golgi compartment (Fig. 3w–y). Together, the image analyses indicate that PGLYRP-1 is expressed throughout the ER and colocalizes with NOD2 at a Golgi-associated compartment.

### GMTriP-K induces interaction of PGLYRP-1 with GEF-H1 and NOD2

**Fig. 4 |:**
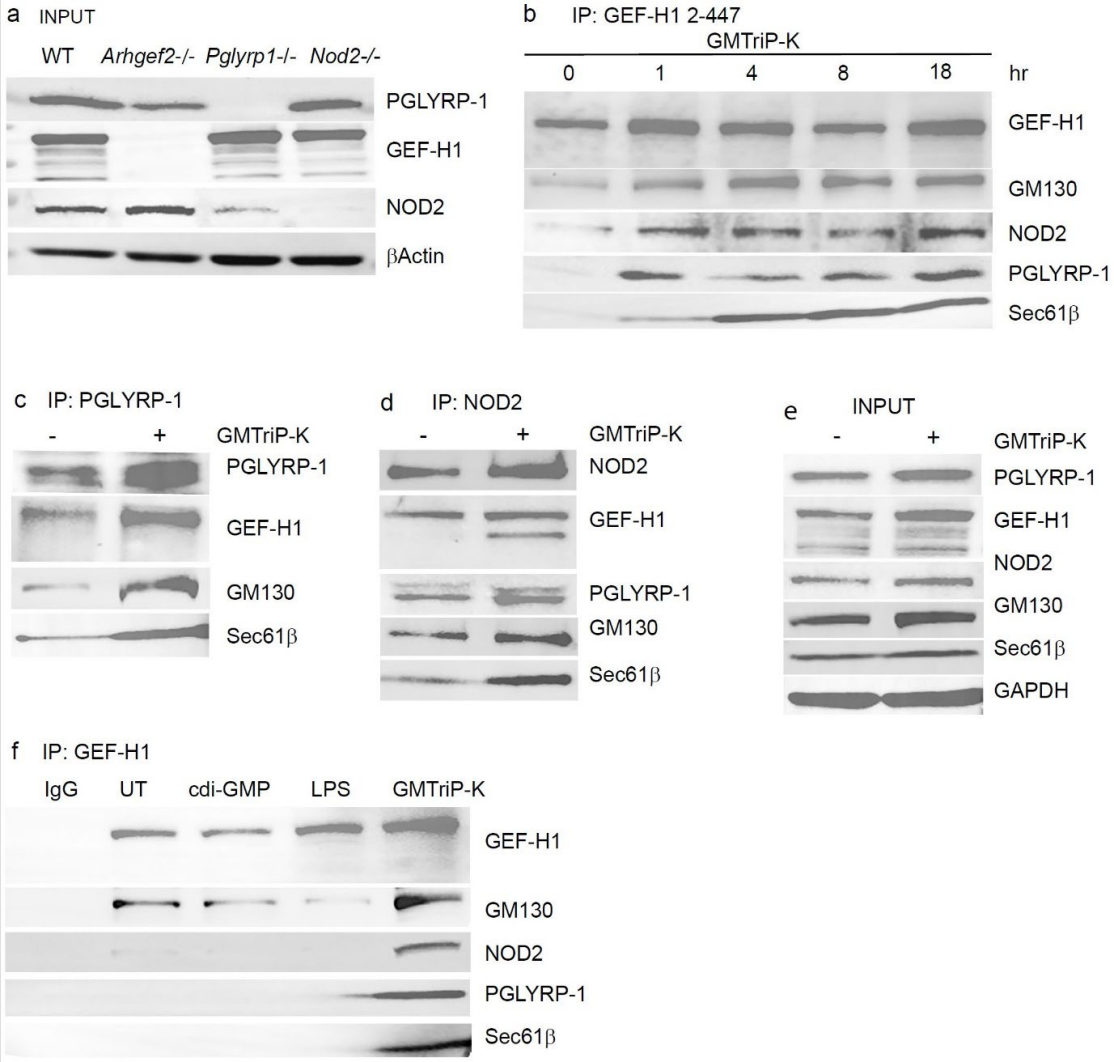
PGLYRP-1 interacts with NOD2 and GEF-H1 in ER- and Golgi-associated compartments **a**, Western blot analysis of protein expression in BMDMs from wildtype, *Arhgef2−/−*, *Pglyrp1−/−*, and *Nod2−/−* mice. **b,** Time course of protein interactions with GEF-H1 after GMTriP-K stimulation of BMDMs. **c,** Analysis of protein interactions with PGLYRP-1 or **(d)** NOD2 after GMTriP-K stimulation of BMDMs. **e,** Input assessment of protein expression for the IPs in **c, d**. **f,** Assessment of protein interactions with GEF-H1 after stimulation with indicated immune stimuli. Representative experiments of at least three repeats are shown.

To better understand the interaction of PGLYRP-1 with the NOD2-GEFH-1 signaling system, we analyzed the expression of PGLYRP-1, NOD2 and GEF-H1 in BMDMs from C57BL/6 wild type, *Pplyrp1*−/−, *Nod2*−/−, and *Arhgef2*−/− mice (Fig 4a). Wild type, GEF-H1- and NOD2-deficient macrophages expressed PGLYRP-1 (Fig. 4a). NOD2 was detectable in wild type and GEF-H1-deficient macrophages but was expressed at lower levels in PGLYRP-1 deficient macrophages (Fig. 4a). The GEF-H1 antibody detected a protein of 125 kDa and three less-expressed isoforms between 90–110 kDa in wild type and PGLYRP-1- and NOD2-deficient BMDMs (Fig. 4a). As expected, the proteins detected by the GEF-H1 antibody were absent in GEF-H1 deficient macrophages (Fig. 4a).

We next examined the interactions between PGLYRP-1, the NOD2/GEF-H1 system and ER and Golgi proteins Sec61β and GM130 during GMTriP-K stimulation. We performed immunoprecipitations of endogenously expressed GEF-H1 at different timepoints after stimulation with 25μM GMTriP-K (Figs. 4b, c). Complexes that contained PGLYRP-1, NOD2 and full length GEF-H1 formed within one hour of stimulation and were maintained over 18 hours (Fig. 4b). Over the stimulation period, GM130 and Sec61β association increased in the complex (Fig. 4b). We were also able to confirm the formation of these complexes after GMTriP-K stimulation by immunoprecipitations with antibodies against PGLYRP-1 or NOD2. (Figs. 4c, d, e).

**Fig. 5 |:**
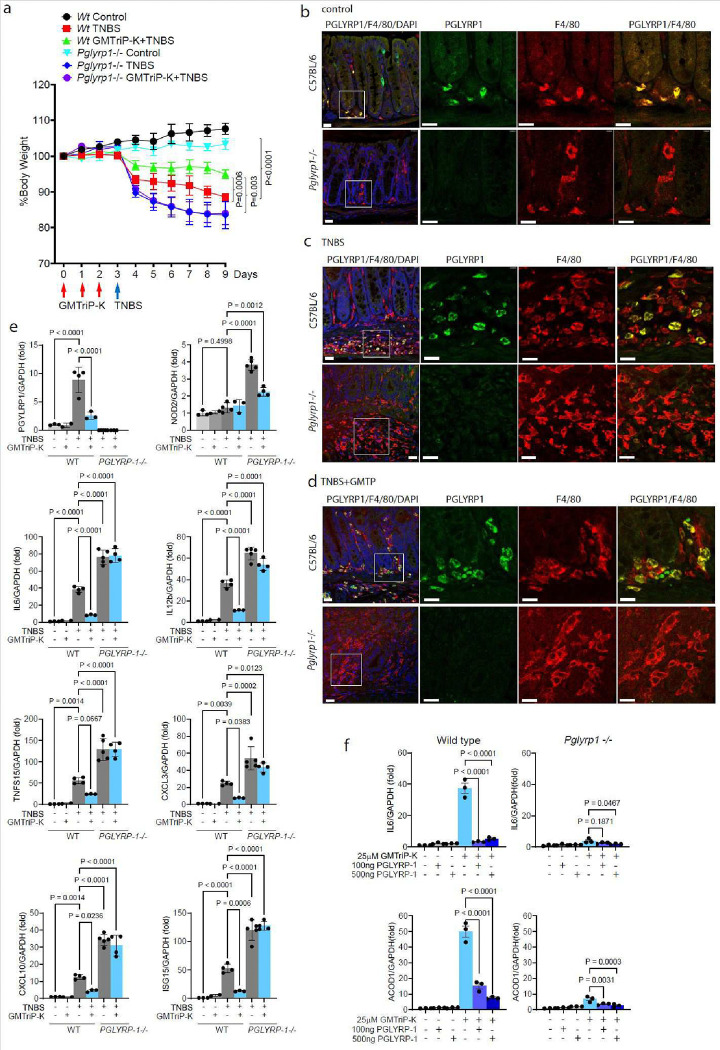
PGLYRP-1 regulates immune responses in TNBS induced colitis **a,** Weight development of wild type and *Pglyrp1*−/− mice during TNBS-induced colitis. Immunostaining of PGLYRP-1 and macrophages (F4/80) in colon sections of wild type and *Pglyrp1*−/− mice before **(b)** after TNBS induced colitis **(c)** and TNBS colitis induction after 3 days of GMTriP-K pretreatment **(d)** at day 9 (bars indicate 25μm). **(e)** qPCR analysis of gene expression in mesenteric lymph nodes of wild type and *Pglyrp1*−/− mice on day 9 of the TNBS and GMTPriP-K treatment protocol. **(f)** qPCR analysis of gene expression in BMDMs from wild-type or *Pglyrp1*−/− mice after stimulation with GMTriP-K in the presence of the indicated amount of PGLYRP-1.

Finally, we found that PGLYRP-1/GEF-H1/NOD2 complexes that contain Sec61β and GM130 formed specifically in the presence of GMTriP-K (Fig. 4f). In these experiments we stimulated macrophages with 25μg /mL bis-(3′-5′)-cyclic dimeric guanosine monophosphate (cdi-GMP), 100ng/mL LPS or 25μM of GMTriP-K and used the N-terminus GEF-H1 antibody to pull down protein complexes. GEF-H1 complexes that contained PGLYRP-1/NOD2 and Sec61β only formed in the presence of GMTriP-K (Fig. 5f). GEF-H1 interacted with GM130 in unstimulated, cdi-GMP and LPS stimulated macrophages and only GMTriP-K stimulation significantly increased GM130 in the complex (Fig. 5f).

Together, our data indicate a new mechanism of PGN recognition in which intracellular PGLYRP-1 interacts with NOD2 and GEF-H1 at the ER and Golgi compartments. GEF-H1 may function as a signaling hub that facilitates PGLYRP-1 signaling during the transition from ER to Golgi associated membrane complexes.

### PGLYRP-1 mediated immune responses regulate intestinal inflammation

To determine the importance of GMTriP-K detection by PGLYRP-1 in the regulation of intestinal inflammation, we studied hapten induced colitis after administration of trinitrobenzene sulfonic acid (TNBS). As shown in Figure 5A, GMTriP-K administration prior to the TNBS challenge protected mice from the weight loss seen in wild type mice during the development of TNBS colitis. Remarkably, *Pplyrp1*−/− mice developed more severe colitis than wild type mice in response to TNBS administration losing more weight much rapidly compared to wild type mice (Fig. 5a). Importantly, pretreatment with GMTriP-K was unable to protect *Pplyrp1*−/− mice from TNBS induced colitis (Fig. 5a).

The assessment of PGLYRP-1 expression in colon sections identified F4/80 positive macrophages in the colon lamina propria (Fig. 5b). It is noteworthy that our antibody was specific as the PGLYRP1 staining was only absent in *Pglyrp1−/−* mice (Fig. 5b). Upon induction of TNBS colitis, the lamina propria of wildtype mice showed inflammatory cell infiltration with increased recruitment of F4/80 positive macrophages that expressed PGLYRP-1 (Fig. 5c). In GMTriP-K pretreatment mice, PGLYRP-1 positive macrophages were also recruited into the lamina propria although inflammatory cell infiltration was less extensive (Fig. 5d). PGLYRP-1 expressing macrophages were found distributed at the base of colon crypts and between muscle layers (Fig 5d). Regardless of pretreatment with GMTriP-K, *Pglyrp1−/−* mice developed severe TNBS colitis with transmural cell infiltration that was characterized by macrophage recruitment (Fig. 5d).

We also investigated the immune system response to TNBS colitis in mesenteric lymph nodes (MLN). Pretreatment of mice with GMTriP-K prevented the induction of PGLYRP-1 expression that increased 10-fold during TNBS colitis in MLNs suggesting a ligand-receptor feedback loop (Fig. 5e). Remarkedly, *Pplyrp1*−/− mice expressed significantly more NOD2 compared to wildtype mice during TNBS colitis (Fig 5e).

Induction of inflammatory genes during TNBS colitis was significantly alleviated after pretreatment with GMTriP-K for 3 days in wild type mice (Fig 5e). We found that PGLYRP-1 expression was significantly increased during TNBS induced colitis (Fig. 5e). Expression of inflammatory chemokines, IL-6 and IL12b, as well as immune regulators, such TNSF15, CXCL3, CXCL10 and ISG15, were significantly reduced (Fig. 5e). In contrast, *Pplyrp1−/−* mice showed an enhanced expression of these inflammatory factors and this enhanced expression pattern did not significantly change after GMTriP-K pretreatment. This is consistent with their increased susceptibility to TNBS colitis that was not mitigated by GMTriP-K pre-treatment. Together, these data suggest that GMTriP-K challenge can provide immune protection to TNBS induced colitis and this protection requires PGLYRP-1.

PGLYRPs can be secreted by immune cells; and we next investigated whether extracellular PGLYRP-1 could 1) regulate GMTriP-K responses in wild type mouse or 2) restore GMTriP-K detection/signaling in *Pplyrp1−/−* BMDMs. We found that soluble PGLYRP-1 inhibited GMTriP-K-induced Il-6 and Acod1 expression in wild type BMDMs (Fig. 5d). However, soluble PGLYRP-1 could not re-establish GMTriP-K detection/signaling in PGLYRP-1 deficient BMDMs (Fig. 5d), (Fig. 5d), suggesting that extracellular PGLYRP-1 bound to GMTriP-K cannot initiate signaling in macrophages. Taken these data together, GMTriP-K facilitated mucosal recovery in colitis through PGLYRP-1-dependent immune regulation.

### PGLYRP-1 dependent gene expression signature is expressed in ulcerative colitis

To determine the involvement of PGLYRP-1 in human inflammatory bowel disease we analyzed the expression of PGLYRP-1 in healthy colon and ulcerative colitis (UC) by immunostaining. We found that a subset of CD68 positive macrophages near the epithelium expressed PGLYRP-1 in the healthy colon lamina propria (Fig. 6a). In addition, granulocytes were identified based on their nuclear morphology and they also expressed PGLYRP-1 (Fig. 6a). In active UC we found an increased recruitment of PGLYRP-1 expressing macrophages throughout the inflamed colon lamina propria with some of these cells infiltrating and crossing the crypt epithelium (Fig. 6a).

We next investigated whether the PGLYRP-1 dependent gene signature induced by was detectable in inflammatory bowel disease. We analyzed the differential transcriptional responses in active (n=6), inactive (n=8) UC and healthy controls (n=6) and clustered genes what we had identified as regulated by GMTriP-K in macrophages (Fig. 6b, Tab. S11). This analysis identified 4 clusters of genes that were down regulated in active UC (Cluster I), genes downregulated in UC but elevated in inactive disease (Cluster II), genes that were upregulated in active UC and inactive UC (Cluster III) and genes that were significantly upregulated in active UC compared to inactive UC or healthy colon (Cluster IV) (Fig 6b, Tab. S12). Cluster IV contained genes that were PGLYRP-1-dependent and GMTriP-K-regulated, including IL1a, IL6, IL12a, CXCL3, MMPs and ARHGEF2 (Tab. S12).

We found that PGLYRP-1, PGLYRP-2, PGLYRP-3 and NOD2 expressions were significantly increased in active UCs compared to inactive UC and healthy colon (Fig 6c). Active UC samples were also characterized by significantly enhanced IL6, IL1b, IFNG, CXCL3 and NFkB2 expression when compared to inactive UC and healthy control samples (Fig. 6c).

To identify the PGLYRP-1 and GMTriP-K dependent gene signature in human immune responses we analyzed the UC data set for genes that were PGLYRP-1-dependent and GMTriP-K-regulated in macrophages. The volcano blot Fig. 6d showed that the GMTriP-K/PGLYRP-1 dependent gene signature was identified among genes that were significantly upregulated in active UC compared to healthy control samples including IL1a, IL1b, IL6, NOD2, SAA1/2, CXCL3/8/10, IFNG, MMP7/10, ISG15, IRF7 and ACOD1 (Fig. 6d Tab. S13). Remarkably, some signature genes were found to be still upregulated in inactive UC compared to the healthy control group (Fig. 6e Tab. S14). While the expression of genes like IL1a, IL1b, IRF7, and ISG15 decreased, genes like NOD2, ZBP1, CARD6, NFkB2, SAA1, MMP7 and CXCL10 were still significantly increased in inactive disease (Fig. 6e Tab. S14).

In conclusion, these data indicated that PGLYRPs play an important part in mucosal immune responses in ulcerative colitis. A subset of CD68 positive macrophages expressed PGLYRP-1 in normal and inflamed human colons. Moreover, we identified a gene signature in active and inactive UC patients which resembles that of the PGLYRP-1 dependent genes that can be regulated by GMTriP-K.

## Discussion

We found that PGLYRP-1 can function as an ER and Golgi associated peptidoglycan receptor for GMTriP-K. Among the human PGRPs tested on the PGN small fragment array, only PGLYRP-1 specifically bound to the disaccharide motif of GMTriP-K. Our study demonstrates that monomers of PGN such as GMTriP-K and its analogues, derived through amidation of lysine and anhydration of muric acid, can bind with PGLYRP-1. Our fragment PGN microarray approach enables the quantification of glycan-protein binding parameters. Other *in vitro* studies suggest binding specificities of PGN to the four mammalian PGLYRPs, ^22^ these studies have largely focused on the third amino acid residue of PGN, which generally differs between Gram-positive and Gram-negative bacteria, with the former using lysine and the latter using diaminopimelic acid. Binding of PGLYRP-1 to monosaccharide PGN units has been suggested, however, in these studies the PGLYRP-1 protein was bound to a surface and surface plasmon resonance (SPR) was used to detect binding ^23^. In agreement with our work, PGLYRP-1 has been suggested to potentially accommodate binding of GMTetP-K ^24^ and showed some specificity for GMTriP-K containing dimers over GMTetP-K dimers ^25,26^. However, these larger dimer fragments are unlikely to enter the cell and are most likely removed/sequestered by secreted PGLYRP-1. Other PGN arrays focused primarily on large oligosaccharides (larger than 1.5 kDa) where all saccharides were bound via the anomeric position of the reducing end of the carbohydrate, omitting glycan recognition ^27^. In our experiments, PGLYRP-1 bound to the array with μM affinity and showed no binding to monosaccharide or peptide fragments, suggesting that this receptor prefers disaccharide moieties over monosaccharide components.

Importantly, here we uncovered that PGLYRP-1 was required for the GMTriP-K induced immune responses in macrophages and not required in GMDiP or MDP induced responses. PGLYRP-1 dependent transcriptional programming was significantly different from that induced by MDP although both pathways required downstream NOD2 and GEF-H1. We did not observe any phosphorylated product when we assayed NAGK for its ability to phosphorylate GMTriP-K, suggesting that the NOD2 response to this compound was a result of PGLYRP-1 signaling rather than the canonical NAGK pathway.

Microscopy and protein enrichment assays indicate that PGLYRP-1 is part of an ER and Golgi-based signaling system, in which PGLYRP-1 interacts with NOD2 and GEF-H1. PGLYRP-1, GEF-H1, and NOD2 formed complexes with of Sec61β and GM130 in the presence of GMTriP-K. Sec61β connects ER membranes to the microtubule (MT) network by directly binding MTs ^28^, indicating a close relationship between MT and ER. Sec61β was also observed in the post-ER compartment ^28,29^. GM130 is a Golgi-forming protein and has multiple functions in autophagy, apoptosis, cell polarity, cell migration, intracellular protein transport and MT formation ^30^.

Our data now indicates that PGLYRP-1 forms complexes that include GEF-H1 as a requirement for signaling. We have previously identified GEF-H1 is an important signaling interactor for the activation of innate immune kinases in the MAVS and NOD2 pathways ^14,31^. The ER is strongly associated with MT through ER tubules, which are constantly formed from existing ER membranes by associating with the growing plus end of MTs or interacting with MT molecular motors ^32^. It will be important to understand the cell type specific molecular mechanism of MT associated GEF-H1 isoforms and cleavage products in membrane trafficking and assembly of PGLYRP-1/GEF-H1/NOD2 complexes and subsequent immune regulation.

Oure data suggests that PGLYRP-1 signaling may occur during the sequential transition/translocation of PGLYRP-1 from the ER, ER–Golgi intermediate compartment (ERGIC), and Golgi. Our proposed model of PGLYRP-1 signaling (graphical abstract) shows similarities with the STING signaling pathway which requires transition of STING from ER > ERGIC > Golgi ^33^.

Remarkably, GMTriP-K pretreatment protected the mucosa from TNBS induced colitis in wild type mice. A similar function has been proposed for MDP although the underlying mechanisms has been unclear ^34^. PGLYRP-1 dependent genes that were induced by GMTriP-K included major immune regulatory cytokines such as IL1a, IL-6, IL-12b and CXCL3, and regulators such as ACOD1 and ISG15. In addition, deletion of PGLYRP-1 resulted in a significant upregulation of NOD2, suggesting that feedback mechanisms exist between different PGN recognition pathways. In this context, an elevated response to MDP may contribute to the severe TNBS colitis in *Pplyrp1*−/− mice. We suggest that PGLYRP-1-mediated signaling is crucial for mucosal protection, expanding the previous identification of PGLYRP-1 as a scavenger for PGN ^6^. Indeed, we found that extracellular PGLYRP-1 can inhibit the response of macrophages to GMTriP-K but cannot restore the signaling in PGLYRP-1 deficient macrophages, indicating that extracellular PGLYRP-1 is a PGN scavenger but may not be involved in intracellular signaling.

We found that PGLYRP-1 was expressed in macrophages in both the mouse and human colon and upregulated during intestinal inflammation. We were able to detect the PGLYRP-1 dependent gene signature that was regulated by GMTriP-K in ulcerative colitis. Unraveling the role of the PGLYRP-1-based PGN recognition system in inflammatory bowel diseases will be important in the context of diseases associated with NOD2 and GEF-H1 variants. The interdependency of PGLYRP-1 signaling, NOD2 and GEF-H1 will require more detailed understanding of this mechanism specifically in Crohn’s disease and other chronic inflammatory conditions where NOD2 is a major risk factor ^1^. It should be noted that ARHGEF2, the gene encoding GEF-H1, maps to a human chromosome 1 locus associated with susceptibility to inflammatory bowel disease ^35,36^.

Our identification of PGLYRP-1 as a receptor for GMTriP-K does not exclude ligand independent functions of PGLYRP-1 in regulating immune responses. Previous studies have shown that deletion of PGLYRP-1 in mice resulted in reduced tumor growth accompanied by an enhanced activation/effector phenotype in CD8^+^ T cells ^37^. PGLYRP-1 deficient mice were also protected from experimental autoimmune encephalomyelitis (EAE) with defects in antigen presentation and alterations in expression profiles of myeloid cells ^37^. Notably, PGLYRP-1 plays a role in Lyme disease and immune dysregulation ^19^.

In summary, we show that PGLYRP-1 is located at the ER and Golgi apparatus and functions as intracellular receptor for monomeric GMTriP-K in conjunction with NOD2 and GEF-H1. Our results show that macrophages expressing PGLYRP-1 are part of immune responses in human colitis and that ligand-dependent PGLYRP-1 signaling promotes mucosal protection in animal model of the disease.

## Methods

Please see key resources list for antibodies, primers and chemicals used (Tab. S15).

### Glycan microarray fabrication, experimentation and data analysis

Glycan microarray fabrication validation, experimentation and data analysis were performed (see Supplementary Methods). For the microarrays used herein, a total of 110 array components were printed in 16 replicate blocks on 3-D hydrogel NHS-activated slides (3-D Hydrogel Coating(H), Schott Minifab, Phoenix, AZ). Following the printing, slides were vacuum sealed and stored at −20 °C until the time of use. Unreacted NHS groups were blocked before use by immersion in blocking buffer solution (50 mM ethanolamine in 100 mM borate buffer, pH 8.5) for 1 h. The slides were rinsed trice with PBST (PBS + 0.05% v/v Tween 20) and then submerged in water for 5 min before drying by centrifugation (1000 RPM for 5 min) then used immediately for incubation. The slides were enclosed in a ProPlate 16-well module (Grace Bio-Labs, Bend, OR) to separate the 16 identical subarrays for different experimental conditions. Recombinant human PGLYRP-1 (R&D Systems, Minneapolis, MN) was applied to the subarrays at various concentrations (typically 10 μg/mL) in PBST, and the slide was sealed with adhesive film and incubated at RT for 18 hours. Arrays were washed thoroughly with PBST and patted dry. Primary anti-PGLYRP-1 (R&D Systems, Minneapolis, MN) was diluted 100-fold in PBST and applied to the subarrays. The arrays were sealed and incubated at RT for 4 hours, followed by washing and drying. Fluorescently labelled secondary antibody (Cy3-IgG, Invitrogen) was diluted 500-fold and applied to each subarray. The slides were sealed and incubated at RT for 1 hour. Arrays were rinsed with PBST, and the 16-well module was removed, and the slide was fully submerged in water for 5 min. The slide was dried by centrifugation (1000 RPM for 5 min) prior to scanning. Slides were scanned using a GenePix 4000B microarray scanner (Molecular Devices, Sunnyvale, CA). Data analysis was performed using GenePix Pro 7 software (Molecular Devices, Sunnyvale, CA). Missing spots were flagged and excluded from analysis. Background fluorescence was subtracted from median fluorescence, and values were averaged for duplicate spots. Data was processed using Excel and GraphPad Prism 9 software. The supporting information provides full data and displays representative images and graphs.

### Mice

All experimental mice were sex-matched at 6–12 weeks of age using protocols approved by the Committee on Research Animal Care at the UT Southwestern Medical Center, Dallas. *Arhgef2−/−* mice were previously described^31^. C57BL/6 wild type, *Pglyrp1−/−, and Nod2*−/− animals were from Jackson Laboratory (Bar Harbor, ME, USA). All animals were bred and housed in a pathogen-free animal facility according to the institutional guidelines.

### BMDMs culture

BMDM cells were generated by flushing bone marrow cells from femurs and tibia of wild type or *Arhgef2−/−, Pglyrp-1−/− and Nod2−/−* mice and red blood cells were removed by using ACK lysis buffer. Then, 70 μm cell strainers were used to filter BMDM cells, followed by resuspension in complete DMEM supplemented with 10% FBS and 0.5% penicillin/streptomycin mixture, and 200 ng M-CSF. BMDM cells were cultured in 10 cm petri dish at 37 °C, 5% CO_2_ for 6 days before experimentation. BMDMs were detached with 0.25% trypsin solution treatment and 1,000,000 cells/well were seeded in a 6-well plate. 25 μM GMTriP-K, GMDiP, or MDP (Invivogen; Cat# tlrl-mdp), 100 ng/mL IFNγ (#575304, Biolegend), 100 ng/mL IFNβ (#581302, BioLegend), or 500ng/mL PGLYRP-1 (#2590-PGB, R&D system) were used to stimulate BMDM cells for indicated duration up to 18 hours. Proteins and RNA were harvested with RIPA lysis buffer or the Qiagen RNeasy Mini Plus kit. HEK 293T cells were purchased from the American Type Culture Collection and grown in DMEM supplemented with 10% fetal bovine serum and 0.5% penicillin/streptomycin.

### RNA sequencing and analysis

Total RNA was isolated from BMDMs derived from wild type, *Arhgef2*−/−, *Pglyrp1*−/−, and *Nod2−/−* mice, or human colon tissue biopsies using an RNeasy Micro kit (Qiagen). Libraries were synthesized using TruSeq Stranded mRNA sample preparation kit from 500 ng of purified total RNA and indexed adapters according to the manufacturer’s protocol (Illumina). The final libraries were quantified using a Qubit fluorometer (Agilent Technologies), and RT-qPCR was performed using the Kapa Biosystems library quantification kit according to the manufacturer’s protocol. Pooled libraries were subjected to 35 bp paired-end sequencing according to the manufacturer’s protocol (Illumina NextSeq 500). The targeted sequencing depth was set at 30 million paired-end reads per sample. Blc2fastq2 Conversion software (Illumina) was used to generate demultiplexed Fastq files. The adaptors were trimmed using Trim Galore (v0.6.4). The trimmed reads were aligned to the human genome (GRCH38) or mouse genome (mm10) using STAR (2.7.3a). Subsequently, the mapped reads were quantified using the featureCounts of the Subread (v1.6.3) package. Genes with low expression (genes with an expression value of zero in more than 30% of the samples) were removed before subsequent analysis. Gene expression was normalized using the Voom method in the R package “limma” (v 3.50.3). Differentially expressed genes were also identified using this software. Gene set enrichment analysis of differentially expressed genes was performed using the R package “clusterProfiler” (v4.2.2). Volcano plots were generated using R package ggplot2 (v3.5.1). Heat maps were generated using R package pheatmap (v1.0.12).

### Real-time quantitative-PCR

Total RNA of BMDMs from wild type, *Arhgef2*^−/−^, *Pglyrp1*^−/−^, and *Nod2*^*−/−*^ mice was isolated using the RNeasy Mini Plus kit (Qiagen). cDNA was prepared from RNA using a PrimeScript RT Reagent cDNA Synthesis Kit (Takara). Real-time qPCR was performed using PowerTrack SYBR Green master mix (Applied Biosystems) with gene specific primers and and relative expressions were calculated using the ΔΔCT method ^38^. Gene expression was normalized to *GAPDH*. All experiments were repeated at least twice. The primer sequences are listed in Table S15.

### Confocal immunofluorescence microscopy

HEK 293T cells were seeded on 8-well-chambered coverslip slides (ibidi GmbH, cat# 80806) at 2.5 × 10^4^ per well, and cells at 60–70% confluency were transfected the next day using Lipofectamine 3000 reagent (Invitrogen) according to the manufacturer’s protocol. Typically, 50–200 ng of total plasmid DNA was transfected into each well without any cytotoxicity. All expression plasmids encoded for full-length proteins, either untagged or C-terminally tagged with Flag epitope or fluorescent proteins. On the day after transfection, the cells were either used for live cell imaging or fixed and stained for immunofluorescence microscopy. For immunocytochemistry, the cells were fixed with paraformaldehyde (Thermo Scientific Chemicals, cat# 047377.9, methanol-free) which was freshly diluted to 4% with concentrated PBS and water. Essentially, cells were fixed for 10 min at 37 °C, washed with PBS, and permeabilized with 0.1% Triton X-100 in PBS for 90 sec. The cells were washed with wash buffer (0.01% Triton/PBS) and blocked with blocking buffer (5% donkey serum 0.01% Triton/PBS) for 1 hour at room temperature. The cells were sequentially incubated with specific primary antibodies and highly cross-adsorbed secondary antibodies in the blocking buffer for a minimum of 1 hour at room temperature. Thorough washes (3 × 5 min) were performed between and after primary and secondary antibody incubation. Optionally, 2–5 min of nuclear staining with DAPI was performed after secondary antibody incubation. Confocal images were captured using the DMi8 microscope system (Leica Microsystems) with a 63X oil lens, with each image having at least 2048 × 2048-pixel resolution. Multicolor imaging channels (four-color) were typically well separated as DAPI, Alexa Fluor 488/EGFP, mOrange/mRFP/Rhodamine Red-X, and Alexa Fluor 647. In addition, each channel was scanned strictly sequentially to avoid crosstalk between the fluorophores. Colocalization studies were carried out in Imaris image analysis software.

### Western blots and immunoprecipitations

BMDM cell extracts were harvested using RIPA lysis buffer (10 mM Tris-HCl, pH 8.0, 1 mM EDTA, 0.5 mM EGTA, 1% Triton X-100, 0.1% sodium deoxycholate, 0.1% SDS, 140 mM NaCl, diluted with dH_2_O) containing protease and phosphatase single-use inhibitor cocktail (Thermo Scientific, Cat#78430). Standard protocols for western blotting were used for SDS-PAGE and wet transfer onto PVDF membranes. Primary antibodies were diluted in blocking buffer (5% milk or 5% BSA) and incubated overnight at 4 °C. Secondary Anti-rabbit-Ig G-HRP (#7074, Cell Signaling) or Anti-mouse-Ig G-HRP (#7076, Cell Signaling) antibodies were used at 1/3000 dilution and incubated for 1 hours at room temperature. Proteins were visualized using enhanced chemiluminescence (Immobilon Western; Millipore). The following antibodies were used in this study: rabbit antibodies against GEF-H1 (ab155785; Abcam,1/1000 dilution), sheep anti-mouse GEF-H1 (#X1089S; Exalpha Biological; 1/1000 dilution), Rabbit anti-Sec61b(D5Q1W) (#14648,Cell Signaling, 1:1000 dilution), Mouse anti-GM130(#610822, BD biosciences), GAPDH (#2118, Cell signaling, 1:500 dilution), PGLYRP-1(#AF2696, R&D system, 1:500 dilution), NOD2 (#PA5–104317, Invitrogen, 1:500 dilution), RB anti-phospho-stat1(Tyr701) (58D6) (#9197, Cell signaling, 1/1000 dilution), RB anti-stat1 (D1K9Y) (# 9172, Cell signaling, 1/1000 dilution), and mouse anti-β-actin (8H10D10) (#3700, Cell signaling, 1/10000 dilution) , Anti-FLAG (#F7425, Millipore Sigma, 1/3000 dilution). For immunoprecipitations BMDMs were lysed on ice for 20 min in Pierce IP lysis buffer (Thermo Scientific, cat#87787). Cell debris was pelleted by centrifugation and the supernatant was then incubated and rotated for 60 min at 4 °C with protein G plus agarose (Pierce Thermo Scientific, cat#20423). Precleared lysates were incubated and rotated at 4 °C overnight with immunoprecipitation antibodies. The protein G agarose beads were added, and the incubation continued at 4 °C for 4 hours. Following three washes with the lysis buffer, the agarose beads were mixed with 1 × SDS sample buffer and incubated at 95 °C for 10 min prior to immunoblotting analysis.

### TNBS induced colitis model

Mice were divided into 6 groups: 1) Wild type control; 2) Wild type TNBS; 3) Wild type TNBS plus GMTriP-K; 4) Pglyrp1−/− control; 5) Pglyrp1−/− TNBS; 6) Pglyrp1−/− TNBS plus GMTriP-K. Each mouse was anesthetized with isoflurane and administered with 3.75mg TNBS (, MilliporeSigma, cat#P2297) in 100 μL 45% ethanol solution by intra-rectum injection via a 3.5 French catheter with a 1 mL syringe. Control mice were administered with the ethanol solution without TNBS using the same technique. Colon and mesenteric lymph node were harvested for histology, immune staining and qPCR at the 6th day post TNBS injection.

### Inflammatory bowel disease tissues.

Human colon tissue samples were collected in the Division of Digestive and Liver Diseases at the University of Texas Southwestern Medical Center under the IRB Protocol Number STU-112010–130,” Registry and Biorepository for the study of Gastrointestinal Inflammatory Diseases”

### Immunofluorescence Staining

Tissue sections were baked for 20 minutes, deparaffinized and rehydrated. Slides were washed with PBS three times for 5 minutes each wash. Citrate buffer pH6 was used as antigen retriever solution. Tissue sections were permeabilized with 0.25% Triton X-100 and blocked with 5% normal donkey serum). After washing the slides with PBS, the sections were incubated overnight in a primary antibody against PGLYRP-1 (R&D Systems, cat#AF2696) with 1:500 dilution and F4/80 Cell Signaling Technology, cat#70076) with 1:300 dilution, in 4 C in a humidified shielded chamber. After one wash with 0.05% Tween/PBS (0.05% PBST) followed by two washes with PBS, the slides were then incubated with secondary antibodies, anti-goat Alexa Fluor 488 (Life Technologies, #A32814TR) and anti-rabbit Alexa Fluor 594 (Life Technologies, #A11037). The secondary antibodies were diluted in blocking buffer and incubated for 1 hour in shielded humid chamber. Slides were washed with 0.05% PBST and PBS. Sections were counterstained with DAPI. Slides were mounted with Prolong Gold Antifade reagent with DAPI (Invitrogen, cat#P36931). The following antibodies used for immunofluorescence staining in this study: rabbit antibodies against F4/80 (Cell Signaling Technologies #70076; 1:1000), CD68 (Cell Signaling Technologies # 76437; 1:1000), Goat antibodies against PGLYRP-1 (R&D system, #AF2696; 1:1000 dilution and #AF2590; 1:1000).

### Quantification and statistical analysis

Statistical analyses were performed using Prism (GraphPad 9) or R (Rstudio). Statistical significance was evaluated by two-tailed unpaired Student’s t-test when comparing two groups; two-tailed paired Student’s t-test for group comparisons; one-way ANOVA for comparing more than two groups; and the Wilcoxon rank sum test for RNA-seq data. Correction methods were labeled in figure legend. p values are indicated. *ns*, not significantly different. Bar graphs show the entire range of values denoting the median.

## Extended data

**Extended data Fig. 1 |:**
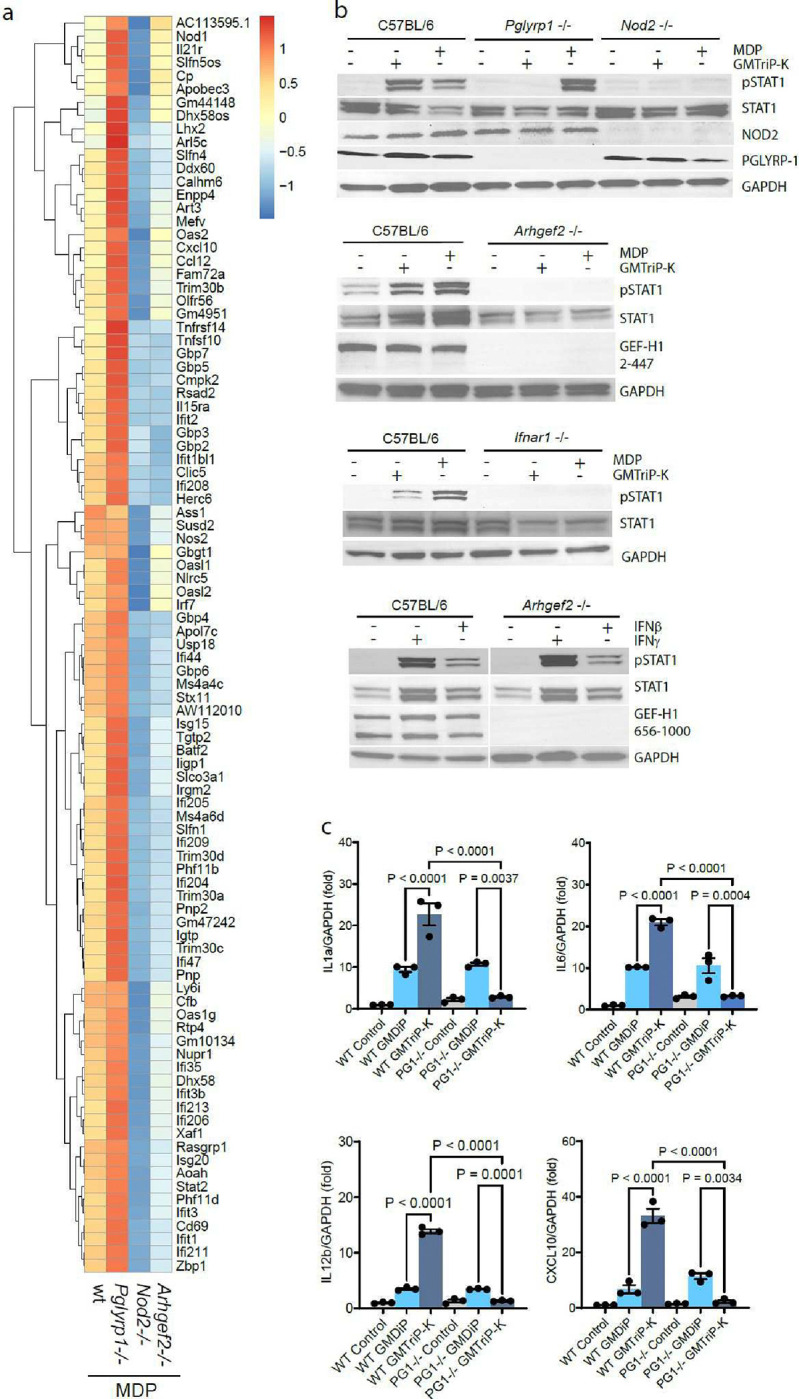
PGLYRP1 is required for type I interferon induction by GMTriP-K **(a)** Hierarchal clustering of MDP induced genes in BMDMs from wild-type, *Pglyrp1*^−/−^, *Nod2*^*−/−*^, or *Arhgef2*^*−/−*^ mice. **(b)** BMDMs from wild type or *Ifnar1*^−/−^ mice were stimulated with 25 μM GMTriP-K or 25 μM MDP for 18 hours and analyzed for STAT1, pathway activation with the indicated phospho-specific antibodies as indicated. **(c)** and **(d)** BMDMs from wild type, *Pglyrp1*^*−/−*^, *Nod2*^−/−^, and *Arhgef2*^−/−^ mice were stimulated with 25 μM GMTriP-K or 25 μM MDP, lysed, and analyzed via western blotting with the indicated antibodies. **(e)** Wild type or GEF-H1-deficient BMDMs were treated with 100 ng/mL of IFNγ or IFNβ for 18 hours, and cells were lysed and analyzed via western blotting with the indicated antibodies. The results represent three independent experiments, and GAPDH was used as loading control. **(f)** pPCR analysis of gene expression in BMDMs from indicated mouse strains stimulated with 25 μM GMTriP-K or 25 μM GMDiP stimulation for 18 hours.

## Figures and Tables

**Fig. 6 |:** The PGLYRP-1 receptor system is regulated in ulcerative colitis **a,** Immunostaining of PGLYRP-1 and macrophages (CD68) in tissue sections of healthy human colon and active ulcerative colitis (bars indicate 25μm). **b,** Hierarchal cluster analysis of genes that were significantly regulated in active, inactive ulcerative colitis and healthy control colons. **c,** qPCR analysis of expression of indicated gene in healthy control n=6), inactive (n=8), and active ulcerative colitis (n=6) samples. **d,** Volcano blot analysis of gene expression comparing gene expression in healthy control and active ulcerative colitis or **(e)** in healthy control and in active ulcerative colitis patients.

## Data Availability

Fastq files of RNA sequencing data will be deposited in GEO.
